# Apicoplast-Resident Processes: Exploiting the Chink in the Armour of *Plasmodium falciparum* Parasites

**DOI:** 10.1155/2024/9940468

**Published:** 2024-05-10

**Authors:** Collins Ojonugwa Mamudu, Mercy Eyitomi Tebamifor, Mary Ohunene Sule, Titilope Modupe Dokunmu, Olubanke Olujoke Ogunlana, Franklyn Nonso Iheagwam

**Affiliations:** ^1^Department of Biochemistry, Covenant University, Ota, Nigeria; ^2^Covenant Applied Informatics and Communication Africa Centre of Excellence, Ota, Nigeria; ^3^Confluence University of Science and Technology, Osara, Kogi, Nigeria; ^4^Covenant University Public Health and Wellbeing Research Cluster, Covenant University, Ota, Nigeria

## Abstract

The discovery of a relict plastid, also known as an apicoplast (apicomplexan plastid), that houses housekeeping processes and metabolic pathways critical to *Plasmodium* parasites' survival has prompted increased research on identifying potent inhibitors that can impinge on apicoplast-localised processes. The apicoplast is absent in humans, yet it is proposed to originate from the eukaryote's secondary endosymbiosis of a primary symbiont. This symbiotic relationship provides a favourable microenvironment for metabolic processes such as haem biosynthesis, Fe-S cluster synthesis, isoprenoid biosynthesis, fatty acid synthesis, and housekeeping processes such as DNA replication, transcription, and translation, distinct from analogous mammalian processes. Recent advancements in comprehending the biology of the apicoplast reveal it as a vulnerable organelle for malaria parasites, offering numerous potential targets for effective antimalarial therapies. We provide an overview of the metabolic processes occurring in the apicoplast and discuss the organelle as a viable antimalarial target in light of current advances in drug discovery. We further highlighted the relevance of these metabolic processes to *Plasmodium falciparum* during the different stages of the lifecycle.

## 1. Introduction

The apicoplast (apicomplexan plastid) is a relict nonphotosynthetic plastid-like organelle present in apicomplexans such as Plasmodium species. It is an unusual organelle proposed to have evolved due to secondary and tertiary endosymbiosis, giving rise to a galactolipid-rich four-membrane organelle. This process involves the phagocytic assimilation of a plastid-containing eukaryote (which originally obtained its plastid via primary endosymbiosis of prokaryotes such as a red alga) by another eukaryote [1–3]. The outer membrane has features similar to a host's endomembrane as a result of phagocytosis. The second outer membrane, also called the periplastid membrane (PPM), is believed to originate from the plasma membrane of the engulfed red alga, while the inner pair of membranes is analogous to the membrane of the chloroplast [3, 4]. Plastids have various metabolic functions in organisms in which they are resident and are mostly photosynthetically active; however, the apicoplast is nonphotosynthetic but plays crucial roles in the survival and development of apicomplexans [3]. Plastids comprise chloroplasts, amyloplasts, chromoplasts, etioplasts, and leucoplasts [3]. The ancestry of apicoplast-harbouring parasites has been linked to three origins: the clades Platyproteum, Chrompodellids, and Apicomplexa, according to phylogenetic and biological investigations [3, 5, 6].

Apicomplexans are obligate parasites classified into five groups: Piroplasmida, Coccidia, Haemosporidia, Cryptosporidium, and Gregarina. These groups of obligate parasites are intracellular parasites except Gregarina [6]. The more popular groups are Haemosporidia (e.g., *Plasmodium* spp.) and Coccidia (e.g., toxoplasma), which have been implicated in malaria and toxoplasmosis. The apicomplexans are responsible for several diseases affecting humans, livestock, wild animals, and invertebrates [4]. Obligate intracellular apicomplexans, including *Plasmodium* spp. and *Toxoplasma gondii,* depend heavily on the apicoplast for *de novo* synthesis of metabolites required for their normal development. This dependence is because, despite the parasite's ability to scavenge these apicoplast-derived metabolites from their hosts or environment, their complex lifecycle and changing environments pose a challenge to obtaining an adequate amount for their normal development at all stages of their lifecycle. Hence, the parasite could depend on the *de novo* synthetic property of the apicoplast for its survival in certain living environments [3, 7]. There is still an ongoing debate on the presence of plastid in the Gregarina; however, it is absent in the cryptosporidium lineage of apicomplexan. The apicoplast is responsible for four major functions: fatty acid synthesis, isoprenoid biosynthesis, Fe-S cluster synthesis, and haem synthesis [8].

The discovery of the apicoplast in malaria parasites has opened up novel prospects in antimalarial drug discovery and development because the organelle is absent in mammals, essential throughout the lifecycle of the parasite, and it possesses nonmammalian metabolic processes, which are critical for parasite growth and development [4, 7]. Several reports have indicated that the apicoplast is an excellent drug target, and more work is currently being done to develop therapeutic interventions with novel mechanisms of action or combination therapies to abrogate apicoplast-resident metabolic processes and diminish the current problem of antimalarial drug resistance [9–11]. Here, we provide an overview of the metabolic processes occurring in the apicoplast and discuss the organelle as a viable antimalarial target in light of current advances. We further highlighted the relevance of these metabolic processes to *Plasmodium falciparum* during the different stages of the lifecycle.

## 2. The *Plasmodium falciparum* Apicoplast

The *Plasmodium falciparum* apicoplast contains genetic material of approximately 50 genes, coding for 30 proteins, rRNAs, and tRNAs, with a size of about 35 kb long [6, 12]. The apicoplast resident genes are responsible for genome maintenance, including processes of replication, transcription, and translation, except for iron-sulphur cluster assembly B (SufB), which plays roles in the sulphur mobilisation (SUF) pathway required for Fe-S cluster synthesis and the ATP-dependent Clp protease subunit C (clpC) gene, whose function is currently undefined but hypothetically believed to be responsible for protein import or as a chaperone hsp70 substitute [6, 13]. The gene products encoded by the nuclear genome required for apicoplast function, called nucleus-encoded apicoplast-targeted (NEAT) proteins, are transcribed in the nucleus, translated in the cytosol, and exported to the apicoplast; however, membrane traversal occurs through a relatively obscure mechanism [3]. The trafficking of nuclear-encoded apicoplast-destined proteins is coordinated by a bipartite canonical signal located at the N-terminal of the protein, which comprises a canonical endoplasmic reticulum (ER)-type secretory signal sequence followed sequel to a plant-like transit peptide [14]. The predominant transport hypothesis for the apicoplast involves the Golgi-independent model, whereby the vesicles emanating from the endoplasmic reticulum convey proteins directly to the apicoplast [14–16].

Apicoplast division and inheritance are important for parasite survival. Further studies on the apicoplast have revealed that it is maternally inherited similarly to the mitochondrial genome (6 kb). The disparity in genome size and lack of copurification during subcellular fractionation differentiated the apicoplast as a distinct organelle from the mitochondria [8]. Nonetheless, both organelles are closely associated during all the stages of the parasite's lifecycle, indicating a strong metabolic reliance between the apicoplast and mitochondria [4, 8].

Recent studies employing exogenous IPP supplementation have been used to study the molecular mechanism involved in apicoplast fission [17]. Autophagy-related proteins have been implicated in autophagy-independent roles, which are critical for apicoplast inheritance and parasite replication during the blood stage of infection [17–19]. During nutrient depletion, *Plasmodium falciparum* ATG8-bounded autophagosomes are destined for the lysosome, where the cargo inherent in the autophagosome is degraded for nutrients. However, during replication, ATG8 has been reported to localise to the apicoplast during the liver and blood stages of parasite development [3, 18]. Walczak and colleagues reported a novel function of ATG8 in *Plasmodium falciparum* in the apicoplast biogenesis [17]. A study by Bansal et al. revealed the importance of the interaction between ATG8 and another autophagy-related protein ATG18. This study showed a novel nonautophagy-related role of ATG18 in controlling ATG8's localisation to the apicoplast and its membrane conjugation [18]. Phosphoinositide kinases (PIKs) phosphorylate phosphatidylinositol to yield phosphatidylinositol 3-phosphate (PI3P), which is required for the cellular localisation of ATG18. However, as the interaction between ATG18 and ATG8 has not yet been validated, it was proposed that PI3P-*Pf*ATG18 is important for *Pf*ATG8 lipidation and may traffic it to the apicoplast [18]. The fission of the apicoplast presents interesting and excellent opportunities for drug development against malaria, though the exact mechanism is still obscure. Recent findings have been encouraging, and elucidating the processes and molecular mechanisms involved will be important in understanding apicoplast biology.

### 2.1. Apicoplast-Localised Housekeeping Metabolic Processes in *Plasmodium falciparum*

Housekeeping metabolic processes are essential in the apicoplast, allowing basic cellular function maintenance and continuity [20]. Because apicoplast division happens over four lipid bilayers, it differs from how bacteria and chloroplasts divide. When the apicoplast separates, it is exactly divided among the merozoite cells that result from this process [21]. Most of the genes in the apicoplast genome are responsible for the transcription and translation machinery of the organelle; however, no DNA replication or organisation genes are present. The apicoplast does not regulate the translation of nuclear-encoded proteins needed, and recent studies reported the accumulation of these proteins in vesicle-like structures in the cytoplasm in apicoplast-negative parasites [22].

Replication begins in the late trophozoite phase and continues into the schizogony phase of the parasite's life cycle, utilizing the D-loop/bidirectional ori mechanism [23]. Proteins associated with replication possess a bipartite N-terminal leader sequence, which destines nuclear-encoded proteins to be post-translationally targeted to the apicoplast via a secretory pathway [24]. Plastidic DNA replication/repair enzyme complex (Prex) is directed to the apicoplast for DNA replication and repair. Primase, helicase, and apicoplast DNA polymerase (apPol) required for template creation, priming, and elongation, respectively, are encoded by the same open reading frame of the Prex gene [25]. This gene is cotranslated as a single polyprotein into the ER lumen and then transported as a polyprotein into the apicoplast [26]. The type II topoisomerases gyrase A (GyrA) and gyrase B (GyrB), which introduce negative supercoils in DNA that are necessary for replication and transcription, are present in the apicoplast [27]. This reduces the strain during the replication process by employing the C-terminal domains of GyrA (which forms a six-bladed propeller) to wrap the DNA with positive handedness and a “DNA clamp” at the N terminus of GyrB (which dimerises when ATP is bound), which seizes another chunk of the same double-stranded DNA (dsDNA) [28].

In contrast with plant chloroplasts with multiple RNA polymerases, apicoplasts only have one RNA polymerase responsible for the transcription of all the genes in the apicoplast genome [29]. The schizont stage is when its expression peaks, coinciding with the replication of the apicoplast genome [30]. The RNA polymerase comprises five subunits: two *α* (rpoA), *β* (rpoB), *β*′ (rpoC), and *ω* (rpoD), of which only the rpoB and rpoC subunits are apicoplast-encoded. The *Plasmodium falciparum* apicoplast employs the polycistronic mode of transcription, and its transcripts (both sense and antisense) exhibit several cleavage sites connected to a UUAUA motif [29]. The apicoplast gene expression machinery is prokaryote-like due to polycistronic mRNA transcripts and 70S ribosomes. The apicoplast genome contains all the rRNA and tRNA genes and several necessary ribosomal proteins, transcription factors, and translation factors [8]. It encodes the open reading frames for the caseinolytic protease clpC, RNA polymerase subunits rpoB, rpoC1, and rpoC2, [Fe-S] cluster protein SufB, and the translation elongation factor (EF-Tu) [31]. The coordinated action of translation initiation, elongation, and release factors is necessary to translate proteins encoded by the apicoplast genome to facilitate the various steps of peptide synthesis in the ribosomes [32].

In the apicoplast, translation starts with forming a ternary initiation complex, consisting of a ribosome with a protein-coding mRNA whose initiation codon (AUG) is base-paired to an aminoacylated initiator tRNA and controlled by the action of initiation factors (IFs) [33]. Elongation begins with the entry of the initiator tRNA into a P-site, which causes a conformational shift and opens the A-site for binding a new aminoacyl-tRNA [30]. The *P. falciparum* apicoplast has UAA as the predominant stop codon [34].

### 2.2. Apicoplast-Localised Nonhousekeeping Metabolic Processes in *Plasmodium falciparum*

The apicoplast is an intracellular organelle that is the target of about 400 nuclei-encoded gene products responsible for several metabolic pathways, including haem synthesis needed for the mitochondrial electron transport chain, the SUF mobilisation pathway for the FE-S cluster synthesis, the fatty acid synthesis II pathway, and the methylerythritol phosphate (MEP) pathway [35]. In addition, the apicoplast performs genome replication, transcription, translation, post-translational modification, and protein turnover [36]. Each of the metabolic functions of the apicoplast is described as follows.

#### 2.2.1. Shemin Pathway

**Figure 1:**
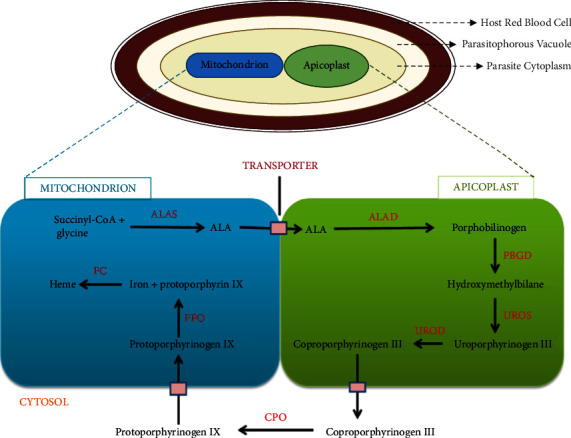
The Shemin pathway. Eight enzymes (depicted in red) resident in three subcellular locations, including the apicoplast, cytosol, and mitochondrion, catalyse this reaction. The substrates and products are depicted in black. ALAS: 5-aminolevulinic synthase; ALAD: 5-aminolevulinic acid dehydratase; PBGD: porphobilinogen deaminase; UROS: uroporphyrinogen III synthase; UROD: uroporphyrinogen decarboxylase; CPO: coproporphyrinogen oxidase; PPO: protoporphyrinogen oxidase; FC: ferrochelatase.

The synthesis of haem occurs via the Shemin pathway, which takes place in three intracellular locations, mitochondria, apicoplast, and the cytosol, and is catalysed by eight enzymes. Following the acquisition of the secondary endosymbiont, the eukaryote was challenged with redundancy in the Shemin pathway, leading to the evolutionary rationalisation of redundancy. The rationalisation of redundancy involved eliminating and substituting similar steps within the pathway, facilitating the collaboration between the mitochondria and apicoplast in haem synthesis [4]. The pathway is initiated by the condensation of glycine and succinyl-CoA in the mitochondria, resulting in the formation of aminolevulinic acid (ALA) by the enzyme ALA synthase, followed by the traversal of ALA to the apicoplast. Four apicoplast-localised enzymes, including *δ*-aminolevulinate dehydratase, porphobilinogen deaminase, uroporphyrinogen III synthase, and uroporphyrinogen III decarboxylase, are involved in the synthesis of coproporphyrinogen III [4, 37]. The subsequent activity of cytosolic coproporphyrinogen oxidase on coproporphyrinogen III leads to the formation of protoporphyrinogen IX, which translocates into the mitochondria and is acted upon by protoporphyrinogen oxidase (PfPPO) and ferrochelatase (FC), resulting in the eventual synthesis of haem (Figure 1) [37].

Haem is an important moiety of biological structures such as chlorophyll and haemoglobin required for binding and carrying small molecules or electrons. The biosynthesis of haem is essential to the malaria parasite, as it is needed as the prosthetic group of cytochromes during the electron transport chain [38]. *De novo* haem biosynthesis is not required in the blood stage but rather during the mosquito stage, making it useful in mosquito transmission [37]. This is possible because the parasite can scavenge haem, and some Shemin pathway enzymes can be scavenged from the hosts. Furthermore, two routes were proposed for haem acquisition from the host during the blood stage, including sequestration and transportation of haem by haem-binding proteins following haemoglobin degradation in the food vacuole or scavenging free haem present in the cytosol of the invaded erythrocyte [37]. The dispensable nature of de novo haem synthesis during the intraerythrocytic blood stage of falciparum malaria decreases the viability of the Shemin pathway as a potential target for antimalarial therapy.

#### 2.2.2. Type II Fatty Acid Synthesis (FAS II) Pathway

Lipids are essential to all living organisms, as they function as building blocks for membranes, for cell growth and differentiation, for maintaining cellular homeostasis, as energy storage molecules, and in post-translational modification processes such as palmitoylation and myristoylation, which control membrane localisation and function of proteins [39, 40]. In light of recent studies, the FAS II pathway is indispensable during the late liver and mosquito stages of the life cycle of *Plasmodium*. However, environmental stress leading to lipid starvation during *in vitro* growth or physiological stress has suggested lipid metabolic plasticity, leading to reliance on the de novo fatty acid synthesis pathway to produce necessary lipid components [6, 41–43]. Malaria parasites obtain the required fatty acids from their host (vertebrate host or mosquito vector) or through *de novo* synthesis (FAS II pathway). The multi-enzyme apicoplast-localised FAS II pathway (also called the dissociative pathway) is distinct from the mammalian FAS I pathway (also called the associative pathway), which takes place in the cytosol and is catalysed by a single giant multidomain enzyme called fatty acid synthase [40, 44].

**Figure 2:**
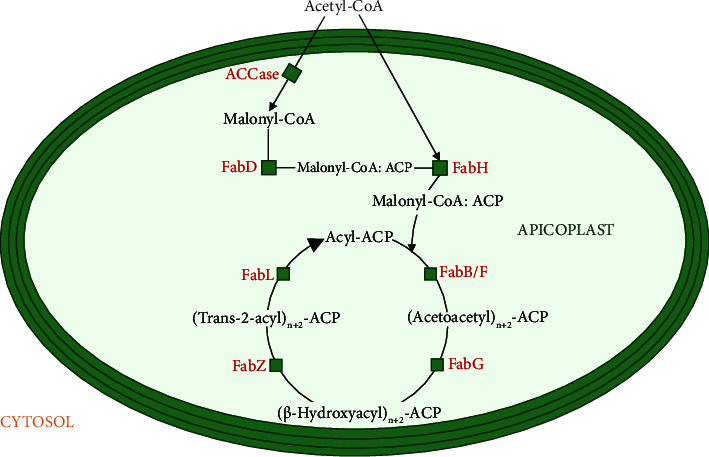
The FAS II pathway in *Plasmodium* apicoplast. Fatty acid synthesis fosters malonyl-CoA formation, which is catalysed by acetyl-CoA carboxylase (ACC). Malonyl-CoA is converted to malonyl-CoA: ACP by malonyl-CoA: ACP transacylase (FabD). *β*-Ketoacyl-ACP synthase III (FabH) catalyses the initiation of the elongation step that leads to the formation of a butyryl-acyl carrier protein (ACP), which is then elongated by condensation with malonyl-ACP to form acetoacetyl-ACP. Acetoacetyl-ACP is reduced to *β*-hydroxyacyl-ACP, which is then dehydrated to trans-2-acyl-ACP and ultimately to acyl-ACP. These steps are catalysed by *β*-ketoacyl-ACP synthase II (FabB*/*FabF), *β*-ketoacyl-ACP reductase (FabG), *β*-hydroxyacyl-ACP dehydratase (FabZ), and enoyl-ACP reductase (FabI), respectively. The *n* + 2 depicts the condensation of two carbons into the nascent fatty acid chain. Repeated cycles of these steps lead to the synthesis of C_14_-ACP.

The FAS II pathway employs six enzymes and the acyl carrier protein (*Pf*ACP), and the process can be partitioned into three stages: preparation, initiation, and elongation (Figure 2). The first phase involves the shuttle of phosphoenolpyruvate (PEP) into the apicoplast from the cytosol via two transporters known as plastidic phosphate transporters (pPTs) resident in the innermost and outermost apicoplast membranes. PEP is then converted to pyruvate via the activity of pyruvate kinase II, which is localised to the apicoplast [9, 45]. The terminal step of the preparation phase involves pyruvate dehydrogenase (PDH), leading to the formation of acetyl-CoA and ATP and reducing the equivalents required for downstream processes [45]. The initiation stage comprises the first committed step in the FAS II pathway, which involves the activity of cytosolic acetyl-CoA carboxylase (ACC) in the carboxylation of acetyl-CoA to malonyl-CoA. Malonyl-CoA is acted upon by malonyl-CoA: ACP transacylase (MCAT; FabD) and converted to a malonyl-acetyl carrier protein (malonyl-ACP) via a Claisen condensation reaction [9, 40, 45].

Ensuing is the initiation of the elongation step by *β*-ketoacyl-ACP synthase III (KAS III; FabH), which condenses malonyl-ACP with acetyl-ACP, leading to the formation of 3-oxoacyl-ACP. Next, 3-hydroxyacyl-ACP is synthesised following the reduction of 3-oxoacyl-ACP by OAR (3-oxoacyl-ACP reductase; FabG). 3-hydroxyacyl-ACP is then dehydrated to form enoyl-ACP by *β*-hydroxyacyl-ACP dehydratase (HAD; FabZ), which is then reduced to a saturated acyl-ACP species catalysed by enoyl-ACP reductase (FabI). The chain length of acyl-ACP is elongated by Claisen condensation with malonyl-CoA, albeit with a different enzyme (FabBF) this time, following subsequent actions of FabG, FabZ, and FabI, ultimately resulting in the synthesis of fatty acids, thus effectively completing the elongation cycle [40, 45].

There are several hypotheses regarding the fate of fatty acids produced via the FAS II pathway, such as a source of the lipid moiety for the glycosylphosphatidylinositol (GPI) anchor required to anchor circumsporozoite protein and other sporozoite proteins to the parasite's plasma membrane, for sporoblast development. However, no study has elucidated the exact fate of FAS II pathway products beyond the apicoplast to date [42, 46]. Recently, Kloehn et al. suggested that the apicoplast-synthesised fatty acid and lysophosphatidic acid were precursors for the bulk lipid synthesis in the endoplasmic reticulum [47]. Nevertheless, two pathways have been expounded as possible fates for the FAS II products within the apicoplast, including the synthesis of lipoate, a cofactor, and fatty acid precursors during membrane lipid synthesis [45]. Lipoate synthesis within the apicoplast is especially important because it is required for its antioxidant properties during the intraerythrocytic stage as there is no apicoplast membrane-resident transporter of lipoate, even if it is scavenged from the host [9, 48]. By targeting the FAS II pathway, drugs can disrupt the synthesis of essential fatty acids necessary to maintain the integrity of the parasite's cell membranes and impede its ability to replicate and survive within host cells [47]. Inhibiting this pathway is, therefore, a promising strategy for developing antimalarial drugs to combat the devastating effects of *Plasmodium falciparum* infection and truncate parasite migration from the liver to the blood asexual stage.

#### 2.2.3. SUF Pathway

Iron is essential to many life processes and acts as an inorganic cofactor in redox and nonredox catalytic processes, including haem and biotin synthesis, repair of DNA, transcriptional control, substrate binding, and nitrogen fixation [49–52]. Despite their numerous functions in living cells, iron is toxic; as such, mechanisms have evolved to obtain, store, and mobilise iron in a less toxic and readily accessible form. Malaria parasites independently partition the sulphur mobilisation pathway for Fe-S cluster synthesis between the apicoplast and mitochondria [50]. Iron and sulphur can coordinate in several forms to form rhombic [2Fe-2S] as found in ferredoxin and cubic [4Fe-4S] Fe-S clusters, or more complex mixed-metal clusters, including [Mo-7Fe-9S] clusters found in nitrogenase [51]. The genes coding for the SUF system are largely located in the nucleus (SufA, C, D, E, and S) and SufB, which is encoded in the apicoplast genome [49]. A recent study was conducted on the indispensability of the FE-S cluster during the mosquito stage of the lifecycle [49]. Also, it has been shown to be relevant for apicoplast maintenance during the blood stage of infection, and disruption of SUF C, D, E, and S functions during the blood stage abrogates infection [50, 53, 54].

**Figure 3:**
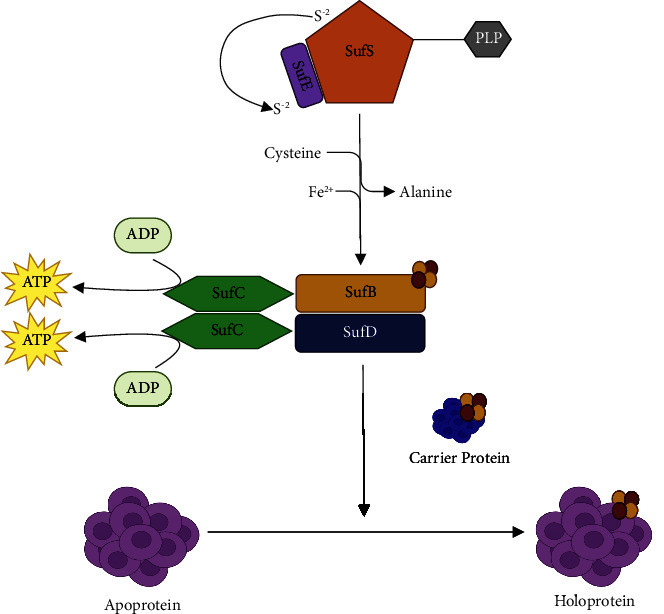
The SUF pathway in *Plasmodium* apicoplast. Cysteine desulfurase (SufS) acts in complex with SufE to mobilise sulphur from L-cysteine to form the Fe-S cluster on the scaffold protein (Suf BCD complex). The Fe-S cluster formed is then transferred via a carrier protein to an apoprotein, ultimately forming a holoenzyme.

The biosynthesis of the Fe-S cluster requires three different groups of enzymes that make up the scaffold protein, including NIF (nitrogen fixation), ISC (iron-sulphur cluster formation), and SUF (sulphur mobilisation) pathways to assemble Fe-S clusters [55]. The ISC pathway provides Fe-S clusters to mitochondria and cytoplasm-resident proteins in *Plasmodium falciparum*. However, the SUF pathway is responsible for the Fe-S cluster biosynthesis in the apicoplast [51]. The biogenesis of the FE-S cluster is not a spontaneous process, as shown in Figure 3. The initial step involves the transference and assembly of iron and sulphur onto a scaffold protein to form a Fe-S complex. This involves the activity of cysteine desulfurase (SufS), which mobilises sulphur from L-cysteine. Charan et al. showed that SufS works in a complex with SufE, which catalyses the speedy mobilisation of the sulphur from SufS to the scaffold protein (SufBCD complex) [49]. Though the exact source of iron is still obscure, it is proposed that an iron-binding homolog YfhJ, in *Plasmodium* spp., is the iron donor instead of frataxin, the proposed iron donor in bacteria [51, 52]. The Fe-S complex forms on the SufBCD complex following the liberation of sulphur from the SufSE complex alongside the presence of iron [51].

The second step involves mobilising the nascent Fe-S cluster to a target apoprotein, which employs the activity of several accessory shuttle proteins, including the homodimeric A-type carrier SufA and NfU-like domain-containing protein (NfU). Hausig et al. reported that SufA is dispensable throughout the lifecycle of *Plasmodium berghei*, and Charan et al. independently reported NfU being the dominant Fe-S cluster transfer protein from the scaffold protein complex in *Plasmodium falciparum* [49, 50]. SufA and NfU have now been reported to be dispensable during the blood stage, leading to conditions compatible with cell death without exogenous supplementation of mevalonate [35]. The SUF pathway is important to the malaria parasite as it provides the Fe-S cluster necessary for the activity of several proteins, including dihydrolipoyl dehydrogenase, glyoxalase I-like protein, ferredoxin, antioxidant protein, glutathione reductase, lipoic acid synthase (LipA), (dimethylallyl)adenosine tRNA methylthiotransferase, and also the methylerythritol phosphate (MEP) pathway enzymes ((E)-4-hydroxy-3-methylbut-2-en-1-yl diphosphate synthase (IspG) and (E)-4-hydroxy-3-methylbut-2-en-1-yl diphosphate reductase (IspH)) [35, 55]. The Fe-S synthetic pathway is important during the sexual stage of the parasite's development; hence, discovering new and potent inhibitors against this stage is important to arrest the parasite's lifecycle and reduce malaria transmission [56].

#### 2.2.4. Methylerythritol Phosphate (MEP)/Rohmer Pathway

**Figure 4:**
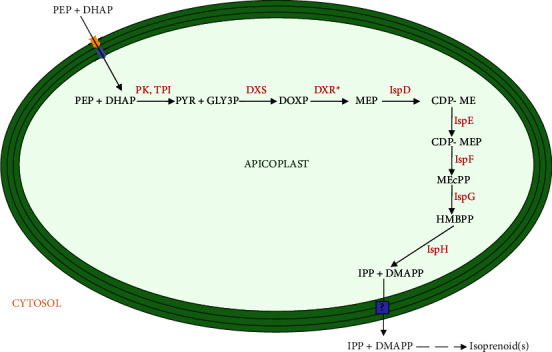
The methylerythritol (MEP) pathway in *Plasmodium* apicoplast. Pyruvate kinase (PK) and triose phosphate isomerase (TPI) catalyse the synthesis of pyruvate (PYR) and glyceraldehyde 3-phosphate (GLY3P), which are the starting materials for the MEP pathway. Seven nuclear-encoded enzymes catalyse this pathway, including DOXS (DOXP synthase), IspC (DOXP reductoisomerase), IspD (2-C-methyl-D-erythritol 4-phosphate cytidylyltransferase (YgbP)), IspE (4-(cytidine-5-diphospho)-2-C-methyl-D-erythritol kinase (CMK)), IspF (2C-methyl-D-erythritol 2, 4-cyclodiphosphate synthase (YgbB)), IspG (4-hydroxy-3-methyl-2-(E)-butenyl-4-diphosphate synthase (GcpE)), and IspH (4-hydroxy-3-methyl-2-(E)-butenyl-4- diphosphate reductase/LytB). Substrate and products: PEP: phosphoenolpyruvate; DHAP: dihydroxyacetone phosphate; DOXP: 1-deoxy-d-xylulose 5-phosphate; MEP: 2-(C)-methyl-d-erythritol 4-phosphate; CDP-ME: 4-diphosphocytidyl-2-(C)-methylerythritol; CDP-MEP: 4-diphosphocytidyl-2-(C)-methylerythritol 2-phosphate; MEcPP: 2-(C)-methyl-d-erythritol 2,4-cyclopyrophosphate; HMBPP: (E)-4-hydroxy-3-methyl-but-2-enyl pyrophosphate; IPP: isopentenyl pyrophosphate; DMAPP: dimethylallyl pyrophosphate. Dxr^*∗*^ (the asterisk signifies that it is the rate-limiting enzyme in the pathway). The mechanism of transport of IPP and DMAPP is unknown.

Isoprenoids are a significant class of biomolecules derived from two 5-carbon isomers, isopentenyl pyrophosphate (IPP) and dimethylallyl pyrophosphate (DMAPP), forming isoprene units, which can undergo modification including cyclisation, oxidation, reduction, and addition, resulting in a repertoire of more than 40,000 naturally found isoprenoids [7, 57]. *Plasmodium* spp. metabolise glyceraldehyde 3-phosphate and pyruvate as precursors to synthesise IPP and DMAPP through the MEP pathway in seven enzymatic steps, as depicted in Figure 4 [58]. Reported evidence on glyceraldehyde 3-phosphate and pyruvate transporters resident in the apicoplast has strongly indicated the apicoplast as the site of isoprenoid biosynthesis [58, 59]. IPP and DMAPP are building blocks for the synthesis of isoprenoids, which are important in *Plasmodium falciparum* in the synthesis of sesquiterpenes and diterpenes for protein prenylation [60], vitamin E synthesis [61], sterols for membrane stability [57, 62], carotenoids [57], ubiquinone as an electron acceptor during pyrimidine synthesis [57], and dolichols for protein modification processes including dolichylation, GPI anchors, and O- and N-linked glycosylation [57]. During the MEP pathway, two ATPs and three NADPH molecules are required to convert glucose to the isoprenoid synthesis precursors [63].

The mevalonate pathway was first reported in 1958 as the classical biosynthetic route for precursors (IPP and DMAPP) of isoprenoid derivatives, and it was initially thought to be the single route to IPP and DMAPP synthesis until a published review by Lichen proposed the possibility of an alternative pathway [64]. However, later studies reveal an alternate pathway in eubacteria and later apicomplexans that bypass mevalonate and employ glyceraldehyde 3-phosphate and pyruvate as starting materials instead of 3-hydroxy-3-methylglutaryl coenzyme A used by the mevalonate pathway [57, 65]. Of all the anabolic pathways that have been associated with the apicoplast, recent discoveries have suggested that the MEP pathway, hence, isoprenoid biosynthesis, is the primary function of the apicoplast during the blood stage of *P. falciparum* lifecycle [3, 47]. Furthermore, Wiley et al. reported that IPP and DMAPP are critical for the development of gametocytes and mosquito stages oocysts and sporozoites [7]. More recently, a novel role of isoprenoid biosynthesis in apicoplast biogenesis via the activity of polyprenyl synthetase, which synthesises long-chain polyprenols necessary for maintaining apicoplast membrane integrity, was reported [66].

Seven nuclear-encoded enzymes targeted to the apicoplast catalyse the MEP pathway including 1-deoxy-d-xylulose 5-phosphate (DOXP) synthase, IspC (DOXP reductoisomerase), IspD (2-C-methyl-D-erythritol 4-phosphate cytidylyltransferase (YgbP)), IspE (4-(cytidine-5-diphospho)-2-C-methyl-D-erythritol kinase (CMK)), IspF (2C-methyl-D-erythritol 2,4-cyclodiphosphate synthase (YgbB)), IspG (4-hydroxy-3-methyl-2-(E)-butenyl-4-diphosphate synthase (GcpE)), and IspH (4-hydroxy-3-methyl-2-(E)-butenyl-4-diphosphate reductase/LytB) [57]. The crystal structures of four out of the seven enzymes of the MEP pathway have been solved, including IspC (PDB Id: 3AU9) [67], IspF (PDB Id: 4C81) [68], and IspH (PDB Id: 4N7B) [69], which have been very useful in current bioinformatics studies for drug development targeted against the apicoplast-localised isoprenoid biosynthesis. Furthermore, all MEP pathway enzymes are promising antimalarial targets [58]. Control of the MEP pathway may involve regulation of enzyme quantity, including transcriptional controls, regulation of enzyme activity, and metabolite availability [57].

## 3. Exploiting the Apicoplast-Resident Processes as an Antimalarial Drug Target

The prokaryotic origin of the apicoplast makes it vulnerable to antibiotics, which have been exploited against *Plasmodium malaria* [40]. The apicoplast houses pathways that are parallel to bacterial pathways, including the FAS II, MEP, and “housekeeping” pathways, which are distinct from analogous mammalian pathways; therefore, antibiotics, including ciprofloxacin, azithromycin, chloramphenicol, clindamycin, tetracycline, fluoroquinolones, aminocoumarin, and rifampicin, have been reported to target this pathway and, hence, have been employed to treat malaria [8, 36, 70]. However, the kinetics of these drugs have made them unattractive in treating acute/complicated malaria due to a phenomenon known as delayed death, in which the introduction of the drug invokes cell death following subsequent merozoite egress and reinvasion of the erythrocyte. Hence, these drugs do not clear parasitaemia immediately except after the second asexual cycle following the introduction of the drug [70]. In addition, repurposing antibiotics for malaria treatment can lead to prolonged use or overreliance, which increases the potential for antibiotic resistance, as observed for folate inhibitors [71–73].

### 3.1. Targeting the Apicoplast Housekeeping Processes

All the apicoplast-resident processes, including housekeeping processes such as DNA replication, transcription, and translation, can be potential antimalarial targets and have been exploited in drug discovery against malaria. The DNA replication of the circular apicoplast genome requires the nuclear-encoded tetrameric DNA gyrase, an ATP-dependent type II topoisomerase that helps resolve topological problems during replication and transcription [28]. Earlier studies reporting bacterial gyrase as a potent antibiotic target have propelled studies into the malaria parasite DNA gyrase as a novel antimalarial target. Several successes have been recorded in targeting gyrase and inhibiting parasite development, including fluoroquinolones (e.g., ciprofloxacin), purpurogallin (PPG), aminocoumarin (e.g., novobiocin), and clindamycin [28, 36, 74]. Also, nuclear-encoded apicoplast DNA polymerase (apPOL) replicates and repairs the apicoplast genome. Recent studies have validated apPOL as a viable antimalarial drug target, with the Malaria Box compound MMV666123 showing promising results [75]. Furthermore, nine small molecules (KU0263501, KU0036696, KU0260920, KU0007309, KU0241474, KU0177470, KU0261556, KU0001071, and KU0271653) have been reported to be potential inhibitors against apPOL [76].

The apicoplast genome codes for RNA polymerase subunits, RpoB, RpoC1, and RpoC2, which make up the RNA polymerase required for DNA transcription to RNA and share homology with bacterial RNA polymerase [36]. Inhibition of this transcription machinery blocks the transcription of the apicoplast genome, which codes for genes responsible for apicoplast maintenance, ultimately leading to cell death. Inhibitors, including rifampin, doxycycline, and spiramycin, have been implicated in abrogating transcription [8, 36].

*Plasmodium falciparum*, just like other apicomplexans, possesses three active translation sites, including the cytosol, mitochondria, and the apicoplast. The latter two have translation machinery similar to that of the prokaryotes, with that of the cytosol being on par with that of the eukaryotes [70]. These active sites individually possess ribosomes, tRNAs, aminoacyl-tRNA synthetases (aaRS), initiation factors, and elongation factors. Conversely, the apicoplast also features 23S and 16S rRNA, 35 tRNAs, 17 ribosomal proteins, the thermo-unstable EF-Tu, and other factors such as *Pf*IF-1, which are encoded by the nucleus and targeted to the apicoplast [36, 70]. Drugs have been developed to truncate the elongation of nascent peptides during translation by targeting the translation machinery (Table 1). These drugs include tetracycline and its derivatives (e.g., doxycycline and tigecycline), chloramphenicol, fusidic acid, kirromycin, amythiamicin A, thiostrepton, micrococcin, lincosamides (e.g., clindamycin), and macrolides (e.g., azithromycin), which abrogate protein synthesis by inhibiting the activity of the apicoplast ribosome and aaRS inhibitors such as glutamate analogue (e.g., Glu-SA), isoleucine anologues (e.g., cispentacin, mupirocin, and icofungipen), tyrosine analogue (e.g., TCMDC-141232), phenylalanine analogue (ochratoxin A), tryptophan analogues (e.g., chuangxinmycin and indolmycin), proline analogue (e.g., halofuginone), and lysine analogues (cladosporin, borrelidin, and febrifugine), which act as competitive inhibitors competing with amino acids for the active site of aaRS [17, 34, 36, 70, 77–84].

Most inhibitors targeting the apicoplast's housekeeping processes are antibiotics that exhibit slow-acting delayed death, characteristic of their use against malaria. Nevertheless, some antibiotics that act immediately are believed to have an off-target effect, resulting in the observed rapid clearance of parasitaemia [70].

### 3.2. Targeting the Apicoplast Metabolic Processes

The apicoplast provides an ideal microenvironment responsible for four anabolic processes such as the MEP pathway for the building blocks for isoprenoid biosynthesis, the Shemin pathway for the synthesis of haem, the SUF pathway for the Fe-S cluster biosynthesis, and the FAS II pathway for fatty acid synthesis. These pathways have presented excellent opportunities for developing antimalarials because they are critical at different time points in the parasite's lifecycle and distinct from similar pathways in their mammalian host. In contrast with inhibitors of the housekeeping processes that seem initially lackadaisical in action against malaria parasites, inhibitors against the nonhousekeeping processes act promptly to impinge parasite survival [85]. Druggable targets, recent advances, and novel inhibitors targeting these nonhousekeeping processes are discussed as follows.

#### 3.2.1. Inhibitors of the Fatty Acid Synthesis

Malaria parasites were previously thought to depend on the host for their fatty acid requirements; however, following the discovery of the FAS II pathway for fatty acid synthesis in the apicoplast in 1998, several earlier research groups worked on developing drugs that impinge on this pathway. Genetic studies, however, elucidated the dispensable nature of the FAS II pathway during the blood stage of infection [45]. This discovery attenuates previous claims of viable inhibitors targeting this pathway and suggests parasite growth inhibition via off-target action. Abrogation of the FAS II pathway is incompatible with parasite cell death during the blood stage. Still, it is critical during the liver stage as the FAS II pathway enzymes in this stage can be targeted as prophylaxis [9]. Although it has faced challenges over the years in its validation as a druggable pathway resident in the apicoplast, promising successes have still been reported (Table 2).

**Figure 5:**
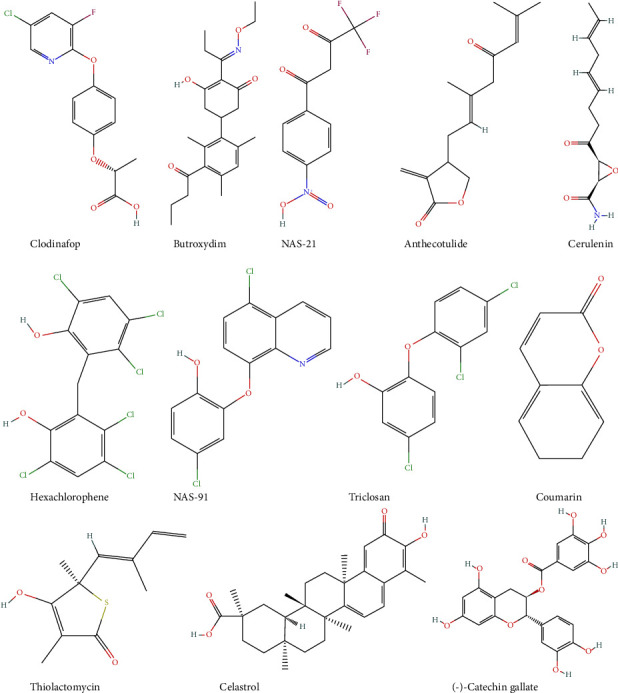
Structures of some FAS II pathway inhibitors.

The FAS II pathway can be grouped into preparatory, initiation, and elongation phases. The preparatory phase involves pPT, pyruvate kinase II (PK2), and PDH. The preparatory phase of the FAS II pathway involves the import of PEP and subsequent conversion to acetyl-CoA and ATP. It is required for the initiation stage of the pathway by employing PK2, PDH, and pPT [45]. These agents of the preparatory stage present promising antimalarial targets. pPT is the only transporter of PEP into the apicoplast resident in the inner and outer membranes of the apicoplast membrane. The inner and outer membrane pPTs have been reported to be critical to parasite growth during the blood, mosquito, and liver stages of the lifecycle, respectively [45]. Acetyl-CoA carboxylase (ACC), the enzyme that catalyses the first committed step of the FAS II pathway, is a known target of herbicides such as clodinafop, butroxydim, and cyclohexanedione (Figure 5) [9, 40].

KAS III/FabH, the enzyme responsible for the Claisen condensation of malonyl-CoA ACP and acetyl-CoA ACP from *β*-ketoacyl-ACP is a target of thiolactomycin (Figure 5). The earlier discovery of the inhibitory activity of thiolactomycin against *E. coli* KAS III prompted studies into their role against the analogous *Plasmodium* KAS III [9]. Interestingly, thiolactomycin has been reported to act against *Pf*KAS III, although with low activity [86]. More importantly, thiolactomycin has greater activity against FabBF, which catalyses the Claisen condensation step during the later elongation step. Thiolactomycin acts as a competitive inhibitor of FabBF by actively competing for the active site with malonyl-ACP, and further studies have led to the discovery of thiolactomycin analogues with greater inhibitory activity [9]. Cerulenin is another important inhibitor of FabBF that has been reported [87]. It acts as an irreversible inhibitor by binding to the cysteine residue of the Cys/His/His catalytic triad of the FabBF enzyme [9]. It can, however, bind to the type I FAS active site, which limits its specificity as an antimalarial [88]. The next enzyme following FabH in the FAS II pathway, FabG, has been reported to be inhibited by a triclosan derivative hexachlorophene, which is very active in inhibiting parasite growth during the hepatocyte stage *in vitro* [40]. FabZ, the enzyme responsible for the synthesis of enoyl-ACP from *β*-hydroxyacyl-ACP via a dehydration reaction, is inhibited by two competitive inhibitors, NAS-75, NAS-79, NAS-91, and NAS-21, in addition to (−)-catechin gallate, which prevents substrate: enzyme interaction by blocking the substrate-binding tunnel [9, 40].

Several inhibitors have been identified against FabI, including coumarin and triclosan, which act as uncompetitive inhibitors (Figure 5). Several advances have been made in modifying triclosan to be a more potent inhibitor, but progress has been slow, and there is still no derivative more potent than triclosan against RabI (reviewed in [9]). Flavonoids have also been reported to be active against FabI, in addition to FabG and FabZ, with hydroxylated flavonoids being the most potent and promiscuous among these Fab enzymes [89]. Computational studies screening flavonoids have also revealed three lead compounds, namely, volkensiflavone, bilobetin, and sciadopitysin, that have good inhibitory activities against FabI [90]. Karioti et al. reported linear sesquiterpene lactones, including anthecotulide, 4-hydroxyanthecotulide, and 4-acetoxyanthecotulide as inhibitors of FabI and FabG [91, 92], and also natural product extracts from Turkish sponge *Agelas oroides* such as 2-hexadecynoic acid, which acts as a competitive inhibitor and blocks the liver stage growth of *P. yoelii* by inhibiting the FabI enzyme [93]. Furthermore, several compounds have been reported to have moderate activity against *Pf*FabI, including Genz-10850, Genz-8575, celastrol, and CHBR5217961 [9]. Natural products have also been explored for their medicinal properties against the FAS II pathway. Natural products include 4-acetoxyanthecotulide, 4-hydroxyanthecotulide, and anthecularin (*Pf*FabI and *Pf*FabG inhibitors), evernic acid and psoromic acid (*Pf*FabI and *Pf*FabZ inhibitors), 3-O-methylquercetin and isowighteone (*Pf*FabI inhibitors), and bromopyrrolohomoarginin and valproic acid (*Pf*FabZ inhibitors) [91, 94–97]. One of the known fates of FAS II products in the apicoplast is the synthesis of a PDH cofactor lipoate. The apicoplast-localised *de novo* synthesis of lipoate employs two enzymes, octanoyl-ACP: protein N-octanoyl transferase and LipA, which are promising druggable targets. However, research is still ongoing to develop potential inhibitors against them [9]. Recently, computational studies employing Petri net analysis on FabD, FabG, and FabF revealed that impinging their activities leads to deadlock in the FAS II pathway [98]. This is important as similar studies could help unravel the complexities of metabolic networks and pinpoint key druggable enzymes for developing new antimalarial drugs.

#### 3.2.2. Inhibitors Targeting the MEP Pathway

**Figure 6:**
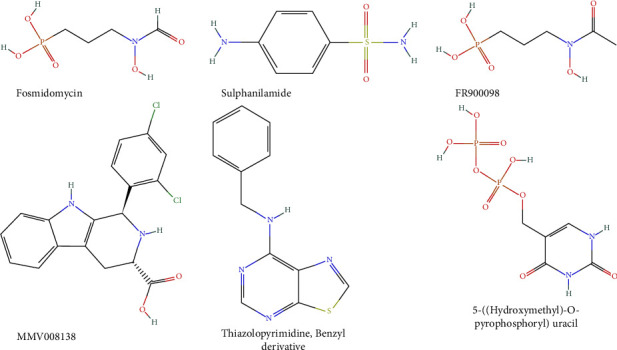
Structures of some MEP pathway inhibitors.

The methylerythritol phosphate pathway comprises seven enzymes that catalyse the conversion of glyceraldehyde 3-phosphate and pyruvate to the building blocks of isoprenoids (IPP and DMAPP). The MEP pathway is responsible for the biosynthesis of isoprenoids, which play critical roles in the parasite's development [99]. Humans biosynthesise isoprenoids via the mevalonate pathway, thereby making the MEP pathway a promising target for novel antimalarials as it is peculiar to the parasite and critical to its survival [57]. The seven MEP enzymes are potential drug targets, and the essential role of isoprenoid biosynthesis in the intraerythrocytic stage of the malaria parasite's lifecycle has prompted research to develop potent inhibitors capable of arresting parasite growth [58]. Currently known inhibitors against this pathway are shown and discussed as follows (Figure 6 and Table 3).

The antibiotics, fosmidomycin (FSM) and FR900098, obtained from *Streptomyces lavendulae* and *Streptomyces rubellomurinus*, respectively, are popular inhibitors of IspC. The inhibition of IspC effectively terminates the availability of downstream precursory metabolites for isoprenoid biosynthesis [58]. Although FSM has poor drug-like properties, clinical trials have shown promising results when co-administered with other known antimalarials, including clindamycin and artesunate [100]. A detailed review of the activity of fosmidomycin and its derivatives is discussed by Patrick and Wang [9, 101]. A new lipid kinase called prenol kinase (*Pf*PolK) has been recently discovered. It is responsible for phosphorylating unphosphorylated isoprenoids (farnesol and geranylgeraniol) [102]. This process helps the parasite survive in an environment where isoprenoids are limited, such as when *de novo* isoprenoid biosynthesis is inhibited by a drug like FSM, and the parasite can scavenge unphosphorylated isoprenoids from the host to supplement its needs in such limited conditions [102]. Although *Pf*PolK is not critical for parasite survival, inhibitors against it could be relevant in developing combination therapy with a novel mechanism of action that could have enhanced efficacy against malaria. Also, *in silico* studies revealed five new compounds, Z-2, Z-3, Z-10, Z-13, and Z-14, and three FDA-approved drugs, aliskiren, ceftolozane, and ombitasvir, that target IspC and have been proposed to be potential antimalarial candidates [103].

The Pathogen Box is a second-generation library released by Medicines for Malaria Venture (MMV) following the prior release of the Malaria Box. It consists of 400 compounds against neglected tropical diseases, of which 125 are targeted against malaria [104, 105]. Docking studies with compounds of the Malaria Box against the IspD have revealed MMV-008138 and its analogues as potent inhibitors, acting via competitive inhibition with the IspD substrate [9, 106]. Reker et al. have also reported 6-amino-7-(1H-benzo[d]imidazol-2-yl)-5-[5-(diethylamino)-1-methylbut-1-yl]-5H-pyrrolo [3,2-b] pyrazine-2,3-dicarbonitriles, a noncompetitive inhibitor of IspD [107]. It is important to note that *Pf*IspD shares homology with human IspD; hence, drug development processes should be mindful of an undesirable inhibition of human IspD [106].

Thelemann et al. reported ortho-bis-sulphonamide as an inhibitor of *Pf*IspF following a screening of 40,000 compounds against IspF from *Arabidopsis thaliana* [108]. Also, thiazolopyrimidine has been shown to act against *Pf*IspF [109]. To date, no potent inhibitors against IspE, IspG, and IspH have been reported to reach clinical trials, although derivatives of diphosphonate, e.g., alkyl phosphate, have been shown to possess inhibitory activities against IspG and IspH and 5-((hydroxymethyl)-O-pyrophosphoryl) uracil as an inhibitor against IspH [9, 58, 110, 111].

#### 3.2.3. Inhibitors Targeting the SUF and Shemin Pathway

Recent studies have strengthened the SUF pathway as a promising antimalarial drug target, which is important during the blood and mosquito stages. Nonetheless, the identification of potent inhibitors against this process is still a work in progress [49, 50, 55]. Table 3 shows a study that reported moderate activity of D-cycloserine against SufS during the asexual blood stage [55]. Given that *P. falciparum* SufS (*Pf*SufS) is not yet fully characterized, it shares a high degree of protein sequence and active site similarity with *Bacillus subtilis* SufS (*Bs*SufS). *Bs*SufS was employed as a model system to elucidate D-cycloserine's reaction mechanism and probe for stronger SufS inhibitors [112]. L-Cycloserine, an enantiomer of D-cycloserine, was reported to be a higher affinity inhibitor of SufS than D-cycloserine [112].

Research targeting the development of inhibitors against Shemin has not progressed into product development despite the druggable targets and earlier reports of potent inhibitors. This is because the Shemin pathway has been discarded as relevant during the clinically relevant phase, i.e., the blood stage of malaria infection; thus, the inhibitors probably showed positive inhibitory activity during this stage due to the off-target effect [70].

## 4. Conclusion

Plasmodium falciparum is responsible for most malaria-related deaths, developing an array of molecular machinery that mediates host-parasite interaction, tropism, and invasion of the human red blood cells [113, 114]. Over the last century, malaria eradication strategies and interventions have been fairly successful, but importantly, it has shown how resilient the malaria parasite can be, as it has co-evolved with each new intervention, and eradication is still a work in progress [115]. The fact that both the housekeeping and nonhousekeeping processes in the apicoplast are distinct from analogous mammalian processes makes them attractive druggable antimalarial targets. Maximising this potential to develop potent antimalarials has been met with limitations, including off-target activity of inhibitors [70], poor drug-like properties of potent inhibitors such as fosmidomycin [100], delayed death [116], drug resistance [117], and an increase in antibiotic resistance [71]. Furthermore, the nuclear genome of *Plasmodium falciparum* is predicted to contain about 5–10% apicoplast targeting signal. However, 70% of these apicoplast gene products are of no known function [118, 119]. The difficulty of developing and implementing reliable screening assays that are adapted to apicoplast biology is one of the primary obstacles to identifying and verifying potential therapeutic targets in the apicoplast pathways. Furthermore, thorough functional characterization of potential drug targets to evaluate their precise role within the apicoplast can be difficult [84, 120].

Despite these limitations, progress has been made in elucidating the apicoplast biology that has played important roles in apicoplast-targeted drug discovery. Chemical rescue using IPP supplementation has been an important tool for studying apicoplast biology [121]. Recent studies employing this tool have led to the discovery of autophagy-related proteins, ATG8 and ATG18, which have been implicated in apicoplast biogenesis, and caseinolytic proteases, ClpC and ClpP, needed for apicoplast segregation, the ATP-binding cassette protein ABCF1, and the membrane transporter DMT2, in addition to studying apicoplast activities in response to treatment responses [17, 18, 70, 122–124]. Furthermore, DNA aptamers are gaining increasing prominence as an alternative therapeutic option against malaria. Recent studies have employed aptamer technology to develop oligonucleotide aptamer-based synthetic antibodies targeting critical apicoplast proteins, in addition to its use as a biomarker for apicoplast-positive parasites and potentials in diagnostic and therapeutic purposes [125, 126]. In light of advancing technologies in bioinformatics, genomics, proteomics, structural biology, and medicine, there has been a significant increase in the identification of inhibitors targeting apicoplast-resident processes; more research is still required to elucidate better the apicoplast biology that will help in novel drug discovery and combination therapies against malaria.

## Figures and Tables

**Table 1:** Druggable housekeeping targets localised in the apicoplast, their function, and inhibitors.

Drug target	Function	Inhibitors	References
DNA gyrase	Type II topoisomerase. Helps resolve topological problems during replication and transcription	Piprofloxacin, purpurogallin, novobiocin, clindamycin	[28, 36, 74]
Nuclear-encoded apicoplast DNA polymerase (apPOL)	Responsible for replicating and repairing the apicoplast genome	MMV666123, KU0263501, KU0036696, KU0260920, KU0007309, KU0241474, KU0177470, KU0261556, KU0001071, KU0271653	[75, 76]
Apicoplast ribosome	RNA translation	Tetracycline, chloramphenicol, fusidic acid, azithromycin	[70, 82]
Aminoacyl-tRNA synthetases (aaRS)	Aminoacyl-tRNA synthesis	Phenylalanine analogue (e.g., ochratoxin A), halofuginone	[17, 84]

**Table 2:** FAS II pathway druggable targets, their function, and inhibitors.

Drug target	Function	Inhibitors	References
Acetyl-CoA carboxylase	Catalyses the first committed step of the FAS II pathway	Herbicides (clodinafop, butroxydim, and cyclohexanedione)	[9, 40]
KAS III/FabH	Condenses malonyl-ACP with acetyl-ACP, leading to the formation of 3-oxoacyl-ACP	Thiolactomycin	[86]
FabBF	Catalyses the second condensation in the elongation step	Cerulenin, thiolactomycin	[9]
FabG	Reduces 3-oxoacyl-ACP to 3-hydroxyacyl-ACP	Hexachlorophene, natural products	[40, 94]
FabZ	Catalyses the dehydration of 3-hydroxyacyl-ACP to form enoyl-ACP	NAS-91 and NAS-21, (−)-catechin gallate	[9, 40, 94]
FabI	Reduction of enoyl-ACP to a saturated acyl-ACP	Coumarin, triclosan, flavonoids, and natural products	[9, 89, 90, 94]

**Table 3:** MEP and SUF pathway druggable targets, their function, and inhibitors.

Drug target (pathway)	Function	Inhibitors	References
IspC (MEP pathway)	Catalyse the synthesis of 2-C-methyl-D-erythritol-4-phosphate (MEP)	Fosmidomycin and its analogues, FR900098	[9, 58]
IspD (MEP pathway)	Catalyses the synthesis of 4-diphosphocytidyl-2C-methyl-D-erythritol (CDP-ME) from MEP	MMV-008138 and its analogues	[106]
IspF (MEP pathway)	Catalyse the synthesis of CDP-MEP into 2-C-methyl-D-erythritol-2,4-cyclodiphosphate (MECP)	Ortho-bis-sulphonamide	[108]
IspG and IspH (MEP pathway)	Catalyse the penultimate and final steps of the MEP pathway, resulting in IPP and DMAPP	Alkyl phosphate	[110, 111]
SufS (SUF pathway)	Catalyses the mobilisation of the sulphur from SufS to the scaffold protein (Suf BCD complex)	D-Cycloserine L-cycloserine	[55, 112]

## Abbreviations

aaRS: Aminoacyl-tRNA synthetases
ACC: Acetyl-CoA carboxylase
ACP: Acyl carrier protein
ALA: Aminolevulinic acid
apPOL: Apicoplast DNA polymerase
clpC: ATP-dependent Clp protease subunit C
DMAPP: Dimethylallyl pyrophosphate
DXP: 1-Deoxy-d-xylulose 5-phosphate
EF-Tu: Translation elongation factor
GPI: Glycosylphosphatidylinositol
IF: Initiation factor
IPP: Isopentenyl pyrophosphate
IspC: DXP reductoisomerase
IspD: 2-C-methyl-D-erythritol 4-phosphate cytidylyltransferase
IspE: 4-(cytidine-5-diphospho)-2-C-methyl-D-erythritol kinase
IspF: 2C-methyl-D-erythritol 2,4-cyclodiphosphate synthase
IspG: (E)-4-hydroxy-3-methylbut-2-en-1-yl diphosphate synthase or 4-hydroxy-3-methyl-2-(E)-butenyl-4-diphosphate synthase
IspH: (E)-4-hydroxy-3-methylbut-2-en-1-yl diphosphate reductase or 4-hydroxy-3-methyl-2-(E)-butenyl-4-diphosphate reductase
LipA: Lipoic acid synthase
MEP: Methylerythritol phosphate
NEAT: Nucleus-encoded apicoplast targeting
PDH: Pyruvate dehydrogenase
PEP: Phosphoenolpyruvate
PI3P: Phosphatidylinositol 3-phosphate
PIK: Phosphoinositide kinases
PK2: Pyruvate kinase II
PPM: Periplastid membrane
pPT: Plastidic phosphate transporter
Prex: Plastidic DNA replication/repair enzyme complex
SUF: Sulphur mobilisation
SufB: Iron-sulphur cluster assembly B
SufS: Cysteine desulfurase.
